# Variability in comorbidites and health services use across homeless typologies: multicenter data linkage between healthcare and homeless systems

**DOI:** 10.1186/s12889-021-10958-8

**Published:** 2021-05-13

**Authors:** William E. Trick, Fred Rachman, Keiki Hinami, Jennifer C. Hill, Craig Conover, Lisa Diep, Howard S. Gordon, Abel Kho, David O. Meltzer, Raj C. Shah, Ed Stellon, Padma Thangaraj, Peter S. Toepfer

**Affiliations:** 1 Health Research & Solutions, Center for Health Equity & Innovation, Cook County Health, Chicago, IL USA; 2 Department of Medicine, Rush University Medical Center, Chicago, IL USA; 3 AllianceChicago, Chicago, IL USA; 4 Alliance to End Homelessness in Suburban Cook County, Hillside, IL USA; 5 Medical Research Analytics and Informatics Alliance, Chicago, IL USA; 6 Jesse Brown Veterans Affairs Medical Center and VA Center of Innovation for Complex Chronic Healthcare, Chicago, IL USA; 7 Section of Academic Internal Medicine Department of Medicine, and Institute for Health Research and Policy, University of Illinois at Chicago, Chicago, IL USA; 8 Center for Health Information Partnerships, Department of Medicine, Feinberg School of Medicine, Northwestern University, Chicago, IL USA; 9 Department of Medicine, University of Chicago, Chicago, IL USA; 10 Department of Family Medicine and Rush Alzheimer’s Disease Center, Rush University Medical Center, Chicago, IL USA; 11 Heartland Alliance Health, Chicago, IL USA; 12 All Chicago, Chicago, IL USA; 13 Center for Housing and Health, Chicago, IL USA

**Keywords:** Homelessness, Substance use, Behavioral health, Privacy-preserving record linkage, Mental health, Health services use

## Abstract

**Background:**

Homelessness is associated with substantial morbidity. Data linkages between homeless and health systems are important to understand unique needs across homeless populations, identify homeless individuals not registered in homeless databases, quantify the impact of housing services on health-system use, and motivate health systems and payers to contribute to housing solutions.

**Methods:**

We performed a cross-sectional survey including six health systems and two Homeless Management Information Systems (HMIS) in Cook County, Illinois. We performed privacy-preserving record linkage to identify homelessness through HMIS or ICD-10 codes captured in electronic medical records. We measured the prevalence of health conditions and health-services use across the following typologies: housing-service utilizers stratified by service provided (stable, stable plus unstable, unstable) and non-utilizers (i.e., homelessness identified through diagnosis codes—without receipt of housing services).

**Results:**

Among 11,447 homeless recipients of healthcare, nearly 1 in 5 were identified by ICD10 code alone without recorded homeless services (*n* = 2177; 19%). Almost half received homeless services that did not include stable housing (*n* = 5444; 48%), followed by stable housing (*n* = 3017; 26%), then receipt of both stable and unstable services (*n* = 809; 7%).

Setting stable housing recipients as the referent group, we found a stepwise increase in behavioral-health conditions from stable housing to those known as homeless solely by health systems. Compared to those in stable housing, prevalence rate ratios (PRR) for those without homeless services were as follows: depression (PRR = 2.2; 95% CI 1.9 to 2.5), anxiety (PRR = 2.5; 95% CI 2.1 to 3.0), schizophrenia (PRR = 3.3; 95% CI 2.7 to 4.0), and alcohol-use disorder (PRR = 4.4; 95% CI 3.6 to 5.3). Homeless individuals who had not received housing services relied on emergency departments for healthcare—nearly 3 of 4 visited at least one and many (24%) visited multiple.

**Conclusions:**

Differences in behavioral-health conditions and health-system use across homeless typologies highlight the particularly high burden among homeless who are disconnected from homeless services. Fragmented and high use of emergency departments for care should motivate health systems and payers to promote housing solutions, especially those that incorporate substance use and mental health treatment.

**Supplementary Information:**

The online version contains supplementary material available at 10.1186/s12889-021-10958-8.

## Background

Homelessness is associated with poor health outcomes attributable in part to a high burden of neglected chronic medical conditions, especially behavioral-health disorders; limited access to routine health care; and, direct complications from being unsheltered [1–3]. Provision of supportive housing for people experiencing homelessness reduces potentially injurious behaviors and likely reduces costs from over-utilization of acute health services in subsets of individuals experiencing homelessness [4, 5]. Despite the advantages of providing housing, public investment in housing solutions is inadequate in the US. Some municipalities, like Los Angeles and Chicago, have established flexible housing subsidy pools to stimulate contributions from healthcare organizations, philanthropies, and health payers that can be allocated to housing and supportive services for vulnerable homeless persons [6].

Although there are reports from other geographic regions describing the impact homelessness has on health services use and outcomes, we believed that a regional evaluation of health services use by homeless individuals may motivate health system leaders and payers to contribute to our local flexible housing subsidy pool to advance population health and reduce the economic burden of care fragmentation [7]. Provision of supportive “wraparound” services requires a fundamental understanding of the needs of homeless individuals, which likely varies by the homeless population being served. Typologies of homelessness can be defined through patterns of shelter use (e.g., chronic, episodic, or transitional); these typologies are associated with varying prevalences of behavioral and medical conditions [8]. Identifying homeless typologies based on shelter use provides important guidance for focused interventions; however, we sought to include an evaluation of health system patients who had no record of receiving homeless services. For our evaluation, we grouped people experiencing homelessness into a different framework for typologies, which was defined by the intensity of homeless services provided—including no homeless services, a group that may be identified during health system encounters [9]. People experiencing homelessness do not uniformly access services for reasons that include accessibility (location), perceived limitations on freedom, prior experiences, or self-reliance and pride [10, 11]. Our evaluation compared individuals who do not access homeless services to those with varying intensities of service receipt.

In 2015, the Patient Centered Outcomes Research Network (PCORnet) was formed [12] to establish a common data model for efficient execution of observational research and clinical trials [13]. At the request of health system leadership for Chicago’s node of the PCORnet network, health system partners engaged the regional Homeless Management Information Systems’ (HMIS) and housing advocacy organizations to evaluate health services use among the homeless in Chicago and suburban Cook County, IL. We developed our data linkage project to answer questions posed by health system leaders, homeless agencies, and housing advocates. We evaluated the variability of health system use; healthcare fragmentation across systems; and medical co-morbidities across the spectrum of homeless typologies.

## Methods

### Setting and data aggregation

We aggregated de-identified data from six health systems in Cook County, IL, including academic medical centers, a large county-operated health system, and a network of Federally Qualified Health Centers. The central data hub (MRAIA Inc., Chicago, IL) joined and de-duplicated de-identified data across the health systems and HMIS. Health systems’ data included all encounters and associated diagnosis codes (ICD10) assigned during calendar year 2016. In the PCORnet common data model, health system encounters are categorized as ambulatory, emergency department, or inpatient (hospitalization) [13]. HMIS is a locally managed information technology overseen by the US Department of Housing and Urban Development to inform homeless policy and decision-making at the federal, state, and local levels. We joined data (October 1, 2015 through September 30, 2016) from two separate HMIS geographies (Chicago and suburban Cook County, Illinois) to health system data. Data were de-identified using a hashed identifier process that enabled central de-deduplication without re-identification [14]. Hashes for patients included first and last name, date-of-birth, social security number, and gender in multiple combinations allowing for incomplete data (for social security number); matches were weighted and required to cross a threshold [14]. Multiple encounters are aggregated into a single Master identifier that accounts for variability in individuals’ identifier(s). To avoid local re-identification, we removed hashed identifiers from the joined dataset before distribution to the study analyst. After human subjects review by our centralized IRB, we received expedited IRB approval.

### Analyses

Health system data included demographics (age, sex, race-ethnicity); ICD10 diagnosis codes (designated as primary or secondary); and, encounter type. HMIS data included homeless service program type encounters (e.g., emergency shelter, permanent supportive housing, etc.). The analytic dataset retained homeless individuals who had received care in a participating health system regardless of whether homeless services had been recorded. Thus, we evaluated recipients of homeless and healthcare services (i.e., present in both databases), and those identified as homeless solely by a health system (ICD10 code = Z59.0). Consistent with the goals of our data linkage and to adhere to the standard of minimum necessary data, data use agreements prohibited inclusion of homeless individuals who had no healthcare encounter.

After consultation with housing advocacy experts, we grouped housing service receipt by type of services received, as follows: *only* stable housing (i.e., permanent supportive housing, rapid rehousing, or safe haven); *only* unstable housing (i.e., emergency shelter, street outreach, or transitional housing); or, both stable and unstable housing services. Since the group that received both stable and unstable services (i.e., a hybrid group) was relatively small (*N* = 809) and dissimilar from the other sub-groups, we did not aggregate these individuals into the stable or unstable housing categories; we present results separately for this “hybrid” group.

We accepted the presence of ICD10 code = Z59.0 on a single encounter as a designation of homelessness. Although individuals may cycle in and out of homelessness, we justified this decision because of the relatively short time frame for our sample and because the homeless diagnostic code for recognition of homelessness has poor sensitivity and thus, the absence of the code does not reliably indicate provision of housing [15].

As specified by data contributors, we created the following age categories: < 21, 21 to 44, 45 to 64, 65 to 74, 75 to 89, and ≥ 90 years of age. Since dates are considered Protected Health Information, data sharing agreements prohibited sharing dates at a level more granular than calendar year; thus, we could not assess temporal relationships between housing and healthcare services.

We used ICD10 codes to evaluate prevalence of chronic conditions (all codes associated with a visit) and reason for healthcare encounters (only primary codes—the code designated as the reason for the health system encounter). The reasons for health service encounters (ambulatory, emergency department, or hospitalization) were calculated using the following two methods of grouping ICD10 codes: 1) We manually aggregated primary diagnoses (represented by ICD 10 codes) for the 25 most common reasons for an encounter (i.e., we grouped: “chest pain, other [R07.89]” with “chest pain, unspecified [R07.9]”; “paranoid schizophrenia [F20.0]” with “unspecified schizophrenia [F20.9]”; and, “Unspecified asthma, uncomplicated [J45.909]” with “Unspecified asthma [J45.901]”); 2) We grouped primary diagnoses for behavioral health conditions using the Healthcare Cost and Utilization Project’s (HCUP) Level 2 clinical classifications schema [16].

We performed all analyses using SAS version 9.4 (SAS Inc., Cary, N.C.) or Stata version 14.2 (Stata Inc., College Station, TX). For comparisons of categorical variables, we used the chi-square test. For comparisons of trends across ordered categories, we used the Cochran-Armitage test for trend. Prevalence rate ratios were calculated using the Cornfield 95% Confidence Interval.

## Results

**Fig. 1:**
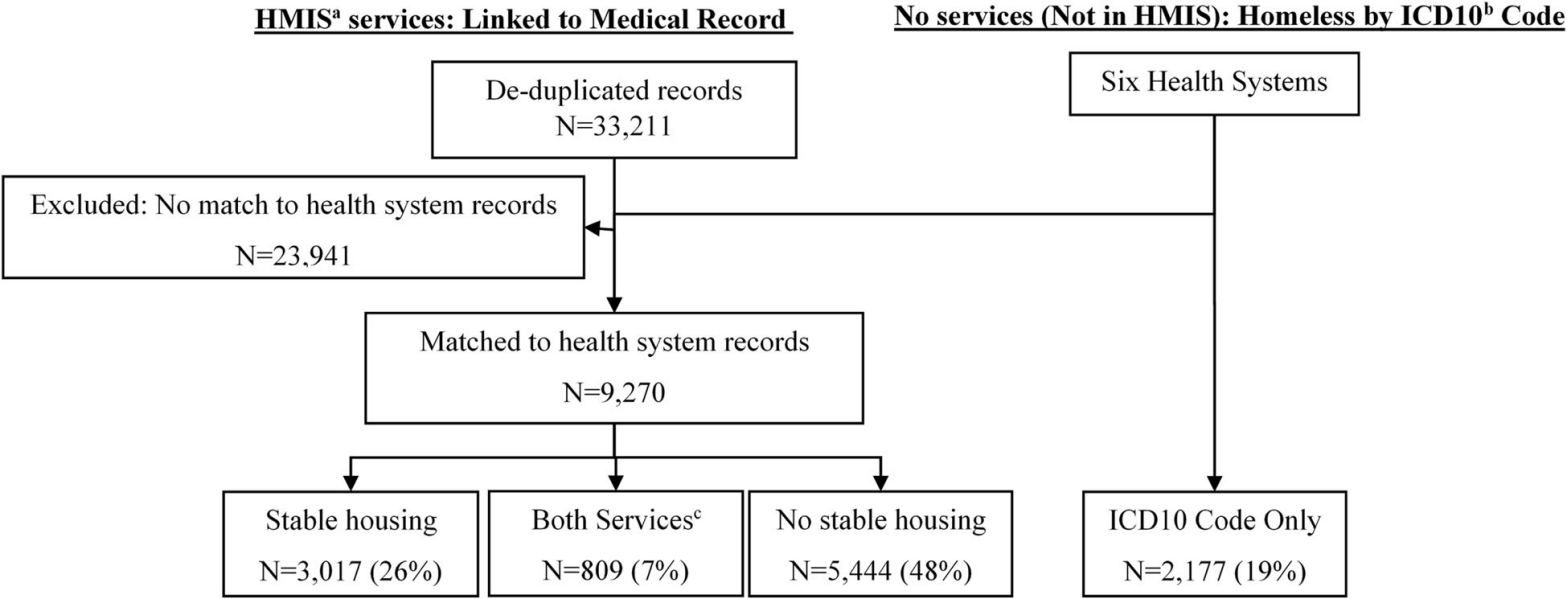
Results of a data linkage between healthcare systems and Homeless Management Information Systems databases. ^a^ HMIS: Homeless Management Information Systems, Chicago and suburban Cook County’s separate systems. ^b^ We used the ICD10 code = Z59.0 to detect homelessness. ^c^ The individual received stable housing as well as services that were not stable housing solutions. Because only year of service was known, the temporal sequence for receipt of services and healthcare encounters is unknown

We identified 11,447 homeless individuals for whom there was at least one healthcare encounter at a participating health system. For most (81%), homelessness was identified through HMIS; however, a substantial minority (*N* = 2177; 19%) were identified as homeless solely through health-system data, Fig. 1. Among literally homeless individuals—those who had not received stable housing—a relatively small percentage were recognized as homeless through health system ICD10 codes (973/5444; 18%). Overall, the majority of homeless individuals were male (62%), non-Hispanic black (71%), and between 21 and 64 years of age (88%), Table 1.

**Table 1:** Characteristics of homeless populations or formerly homeless individuals who had received care at one or more of the six participating health systems, Chicago and suburban Cook County, Illinois

	Homeless services received						No homeless services	
	Stable Housing						ICD10 code only	
	Yes		Hybrid		No			
	*N* = 3017		*N* = 809		*N* = 5444		*N* = 2177	
	N	%	n	%	n	%	n	%
*Age category, years*								
≤ 20	97	3.2*	35	4.3	177	3.2*	97	4.5
21 to 44	765	25.4*	229	28.3*	2177	40.0*	769	35.3
45 to 64	1882	62.4*	485	60.0*	2699	49.6	1042	47.9
65 to 74	162	5.4*	33	4.1*	198	3.6*	151	6.9
75 to 89	16	0.5*	< 10	< 1.0*	44	0.8*	43	2.0
≥ 90	39	1.3	< 10	< 1.0	46	0.8	27	1.2
Missing	56	1.9	19	2.3	103	1.9	48	2.2
*Race*								
Non-Hispanic black	2335	77.4*	616	76.1*	3857	70.8*	1199	55.1
Non-Hispanic white	331	11.0*	98	12.1*	895	16.4*	526	24.2
Hispanic	123	4.1*	35	4.3*	286	5.2*	207	9.5
Non-Hispanic Asian	16	0.5	< 10	< 1.0	30	0.6	12	0.6
Other/unknown	212	7.0*	53	6.6*	376	6.9*	244	11.2
*Sex*								
Male	1633	54.1*	498	61.6*	3484	64.0*	1484	68.2
Female	1334	44.2*	299	37.0*	1919	35.2*	679	31.2
Missing or other	50	1.7	12	1.5	41	0.8	28	1.3
*Homeless service received, stable*^*a*^								
Permanent supportive housing	2734	90.6	411	50.8	–	–	–	–
Rapid rehousing	287	9.5	481	59.5	–	–	–	–
Safe haven	27	0.9	19	2.4	–	–	–	–
*Homeless service received, not stable*^*a*^								
Emergency shelter	–	–	496	61.3	4575	84.0	–	–
Transitional housing	–	–	230	28.4	827	15.2	–	–
Street outreach	–	–	234	28.9	656	12.0	–	–

*Abbreviations: HMIS* Homeless Management Information System

Values representing less than ten individuals reported as < 10, all of which had a percent < 1.0

* Statistically significant difference at *P* < 0.05 significance level, by the chi-square test. No homeless services is the referent group. All comparisons were 2X2 tables; i.e., each variable across each homeless services column was cross tabulated with the referent group

-- Symbol for a value of zero; by definition, these cells are zero

^*a*^ Sums to > 100% because many individuals received services across different categories

**Fig. 2:**
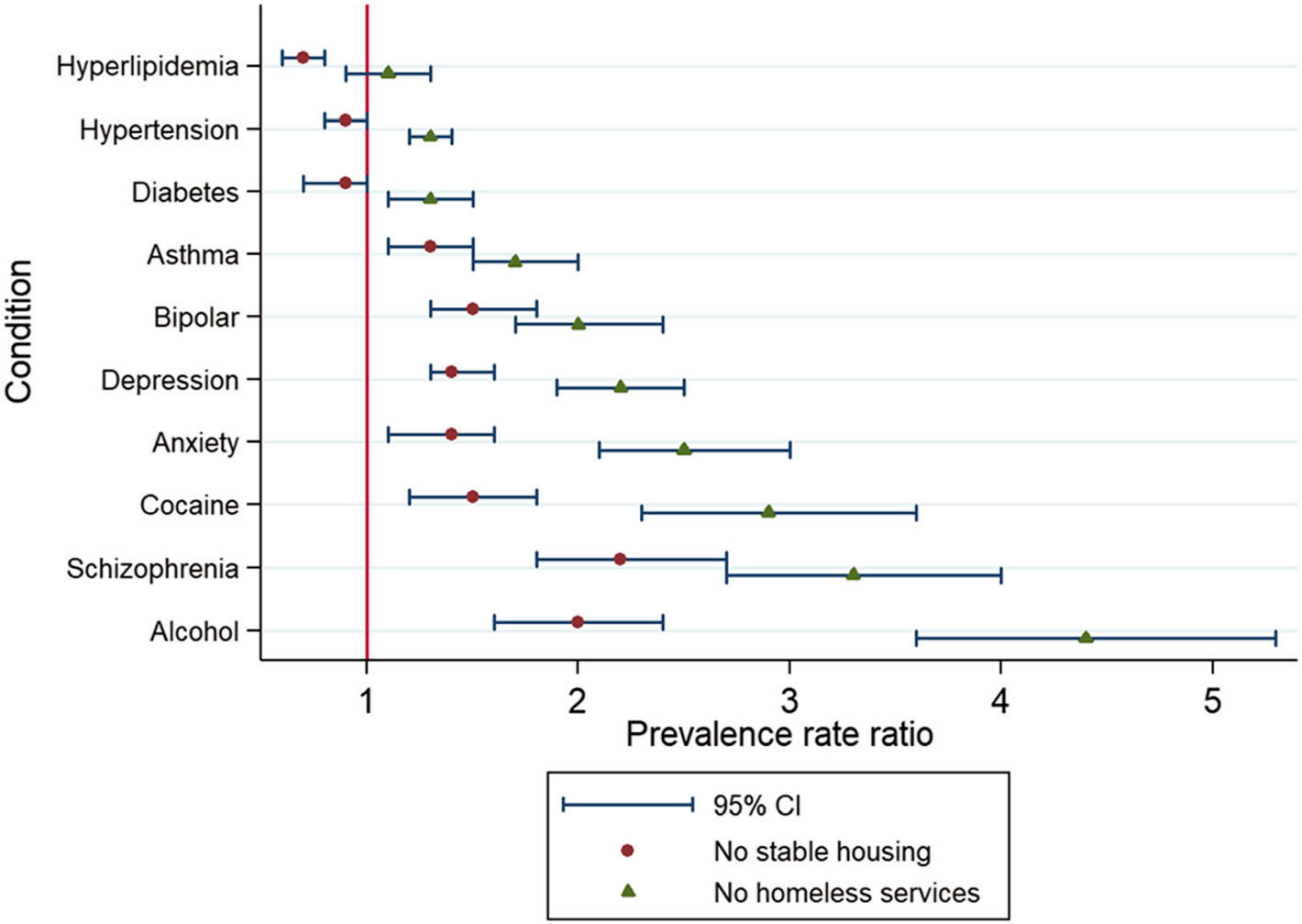
Prevalence of chronic conditions across homeless typologies. Referent group (vertical line) represents stably housed individuals. For schizophrenia, we grouped several diagnostic codes using a narrow definition; i.e., ICD 10 codes F20.X.^13^. ^a^ Homeless by health system ICD10 code only; i.e., no homeless services received

For patients recognized as homeless solely by the health system, there was a higher burden of the most common chronic conditions for nine of the top ten conditions—all but hyperlipidemia, Fig. 2. Compared to those who were stably housed, the prevalence of behavioral health conditions was also more common among those not stably housed, but the differences were not as dramatic, Fig. 2 & Additional File 1.

Across the following four typologies of homelessness ordered by intensity of housing services provided: 1. stable housing only, 2. Both stable and unstable housing, 3. Unstable housing only, 4. No homeless services, there was a monotonic increase in the likelihood of having an emergency department visit or hospitalization. Individuals who had not received homeless services were more likely to have received care in an emergency department rather than an ambulatory visit, and nearly as likely to have been hospitalized, Table 2. Regarding care fragmentation, those without receipt of homeless services had visited more health systems, particularly for emergency department visits; they were approximately four-fold more likely to visit multiple emergency departments compared to those stably housed, Table 2.

**Table 2:** Healthcare services received during the project period, stratified housing services received

	Stable Housing							No homeless services		
	Yes		Hybrid		No			ICD 10 code only		
	*N* = 3017		*N* = 809		*N* = 5444		*P*-value^a^	*N* = 2177		*P*-value^a^
	n	%	n	%	n	%		n	%	
**Visit type**										
Ambulatory	2252	75	649	80	3616	66	< 0.001	1195	55	< 0.001
Emergency room	1165	39	388	48	3436	63		1599	73	
Hospitalization	406	13	139	17	1004	18		1025	47	
**Fragmentation**										
*# of facilities visited*										
1	2132	71	543	67	3546	65	< 0.001	1319	61	< 0.001
2	718	24	189	23	1343	25		560	26	
3	141	5	59	7	396	7		224	10	
≥ 4	26	1	18	2	157	3		74	3	
*# of EDs visited*										
0	1852	61	421	52	2008	37	< 0.001	578	27	< 0.001
1	1007	33	302	37	2527	46		1082	50	
2	141	5	57	7	664	12		355	16	
3	10	0.3	25	3	171	3		121	6	
≥ 4	7	0.2	4	0.5	74	1.4		41	2	

*Abbreviations: ED* Emergency Department

^a^ *P*-values calculated with stable housing as the referent group. The P-values were < 0.001 for both the hybrid and no stable housing groups. For statistical tests of care fragmentation, we used the Cochran-Armitage test for trend across ordered categories

Reasons for emergency department visits varied between homeless typologies; the top ten reasons for patients *without stable housing or without receipt of homeless services* included suicidal ideation, schizophrenia or auditory hallucination, and foot pain, Table 3 & Additional File 2.

**Table 3:** Primary diagnoses for emergency department *visits* stratified by receipt and type of homeless services, Cook County, IL

	Stable Housing						No homeless services		
	No			Yes					
	*N* = 12,490			*N* = 2712			*N* = 8313		
	n	%	Rank	n	%	Rank	n	%	Rank
*Primary diagnosis*									
Prescription refill	852	6.8*	1	137	5.1	2	265	3.2*	6
Chest pain, unspecified	765	6.1*	2	201	7.4	1	623	7.5	1
Alcohol abuse with intoxication	652	5.2*	3	58	2.1	10	403	4.8*	2
Administrative exam	634	5.1*	4	84	3.1	5	338	4.1*	5
Suicidal ideation	571	4.6*	5	44	1.6	12	364	4.4*	3
Asthma, unspecified	552	4.4	6	123	4.5	3	209	2.5*	7
Schizophrenia or auditory hallucinations	372	3.0*	7	14	0.5	37	183	2.2*	10
Low back pain	334	2.7	8	79	2.9	6	354	4.3*	4
Abdominal pain, unspecified	313	2.5	9	72	2.7	7	208	2.5	8
Foot pain	287	2.3*	10	18	0.7	28	185	2.2*	9

Statistical significance testing performed using the chi-square test with stable housing set as the referent group. All comparisons were 2X2 tables; i.e., each variable across each homeless services column was cross tabulated with the referent group

*P < 0.01 for tests of statistical significance

When primary diagnoses were grouped into behavioral health condition HCUP categories (alcohol, illicit substance use, mood, or psychotic disorders), stably housed individuals were much less likely to seek emergency department care or be hospitalized for each of these categories.

## Discussion

Through data linkage between a regional consortium of a national clinical data research network (PCORnet) and Homeless Management Information Systems (HMIS), we evaluated patterns of health services use, care fragmentation, and diagnoses across the following discrete homeless typologies: 1) stable housing, 2) both stable and unstable housing, 3) no stable housing, and 4) no documented receipt of homeless agency services. Notably, individuals with no documented receipt of homeless services were more likely to be Hispanic and in the youngest age category, and to have fragmented health care, especially for emergency department visits. In fact, those who had not received homeless agency services were more likely to have had an emergency department rather than ambulatory visit. Individuals who had not received homeless services had a higher documented burden of comorbidities, especially for behavioral health conditions, such as schizophrenia, and mood, alcohol, or illicit substance use disorders.

Emergency departments are known to be a common source of healthcare service delivery for homeless individuals [17, 18]. We expand on these prior observations by documenting the strong and graded association between housing status and the frequency and fragmentation of emergency department encounters. Most individuals who were stably housed had visited zero, or only one, emergency department; whereas those without stable housing or who had not received homeless services were likely to receive care at multiple emergency departments. By comparison, the Centers for Disease Control and Prevention estimates that 18% of adults in the US visited the emergency department during calendar year 2014—versus nearly two of three individuals without stable housing in our project’s population [19]. Homeless individuals use emergency departments rather than ambulatory visits to access healthcare because of limited access to routine care—in part due to lack of insurance (barriers persist despite Medicaid expansion) and lower levels of instrumental support [20, 21].

When evaluating behavioral health conditions, individuals without stable housing were more likely to have had an emergency department encounter or hospitalization for each HCUP category. For specific diagnoses leading to an emergency department encounter, it was notable that behavioral health conditions (alcohol abuse with intoxication, suicidal ideation, and schizophrenia) were among the top ten diagnoses for homeless individuals without stable housing. In contrast, none of these conditions are among the top ten reasons for an emergency department encounter for the general population. Rather, the top reasons for the general population, in descending order, are as follows: abdominal pain, sprains/strains, superficial injury, nonspecific chest pain, back problems, urinary tract infection, skin and subcutaneous tissue infection, extremity and other open wounds, and upper limb fracture [22]. Of particular interest, one of the top ten reasons for a homeless individual without stable housing to visit the emergency department was for evaluation of foot pain, which was three-fold more likely compared to individuals who had stable housing. Foot problems, especially pain, are common among the homeless, likely resulting from poor foot hygiene, ill-fitting shoes, the requirement to walk for transportation, and prolonged exposure to inclement weather [23].

The most striking demographic difference revealed was that individuals recognized as homeless by a health system but who had not received homeless services were over twice as likely to be Hispanic. We speculate that some Hispanic patients may be reluctant to seek homeless agency services from governmental or charitable organizations that require a registration process, or they may have registered using pseudonyms. Reluctance to seek services may relate to a cultural proclivity for individual self-sufficiency or, for those without secure legal status, fear of deportation [24, 25]. Also, individuals who had no documented receipt of homeless services, were more likely to be in the youngest age category. Relatively young homeless individuals are likely more physically able to cope with the burden of homelessness. Also, there may be reduced awareness of services among the younger homeless population. Clinically, patients coded as homeless without receipt of homeless services had a higher burden of the most common chronic conditions; relative differences were greatest for behavioral health conditions, such as mood and substance use disorders. Currently, most health systems do not assess homelessness comprehensively or according to standardized definitions [26]. Consequently, homelessness documented in clinical records and recorded as a diagnostic code, is likely to denote particular manifestations of the homeless phenomenon. In particular, clinicians are likely to record homelessness as a diagnosis for those who need shelter on discharge and for those most severely impacted by homelessness.

The co-occurrence of psychosocial conditions with homelessness is well recognized, but the higher prevalence of behavioral health conditions among unstably housed homeless persons compared to stably housed homeless persons, suggests that psychiatric illness is a barrier to stable housing that treatment alone may not adequately mitigate [27]. The fragmented utilizers of emergency departments, often with psychiatrically comorbid conditions, represent a highly vulnerable homeless subgroup that were less likely to be represented in the HMIS databases. Our findings may indicate the utility of healthcare data for capturing individuals experiencing unsheltered homelessness and reinforce the need to improve documentation of homelessness into homeless information systems by including healthcare organizations in the systematic assessment of homelessness and seamless initiation of needed services. Integrating cross-sector data systems inclusive of behavioral health, care coordination, and tenancy support can fully capitalize healthcare organizations’ capacity to meaningfully address homelessness, a critically important determinant of health [28, 29].

The utilization of health services was markedly different across the typologies of homelessness. The intermediate intensity of emergency department and hospital utilization among the homeless receiving both stable and unstable housing services, more than the stably housed but less than those without housing services, suggests reductions in utilization attributable at least in part to stability in housing. Other studies have found reductions in healthcare utilization due to the salutary benefits of permanent supportive housing [30]. However, variations in health services utilization in this observational study likely indicate both differences in homeless persons’ engagement with homeless services as well as benefits conferred through stable housing. As healthcare organizations learn to recognize the heterogeneity of homelessness, likely through routine assessments, a needs-targeted approach may more effectively assign housing and supportive resources.

There are several cautions in the interpretation of our findings. Matching individuals is challenging, especially in our target population [31, 32]; undoubtedly, mismatches occurred. The use of administrative codes is hindered by problems of accuracy [33, 34]. The presence of the homeless code in a patient record may indicate either a readily apparent case of homelessness or a detail-oriented, comprehensive coder or clinician, which also may increase the likelihood of coding other chronic conditions. Dichotomized categories of stable vs. unstable housing is not always clear in the case of transitional housing, which can span services similar to emergency shelter through longer stay housing units. We did not capture chronicity or periodicity of homelessness, which is important for identifying previously described homeless typologies [8]. Also, the temporal relationship between housing and healthcare services could not be assessed as dates were not shared, complicating interpretation of the complex interplay between comorbid behavioral health conditions, amenability to housing interventions, and health services use. Misclassification bias may have resulted due to variations in coding practices between encounters and health system sites and by frequency of healthcare utilization. Care fragmentation is a common feature of Chicago’s fragmented healthcare market and may not be directly generalizable to all settings. Medical services are provided to the homeless by mobile health clinics and such encounters would not be documented. Finally, as a cross-sectional observational study, causality and the magnitude of the effect of stable housing on receipt of healthcare services is uncertain.

## Conclusions

As the interoperability of data systems matures and the value of data sharing becomes increasingly recognized, we expect that networks of data systems such as PCORnet will provide valuable opportunities to glean novel insights. Our project is a model for leveraging the value of data linkages across health sectors; we demonstrated how a regional collaboration between health systems and nonprofit social service entities could overcome technical and privacy concerns to generate knowledge helpful in advancing progress toward addressing a critical social determinant of health – housing for homeless individuals.

## Supplementary Information

**Additional file 1.**

**Additional file 2.**

## Data Availability

The datasets generated and analyzed during the current study are not publicly available due to restrictions on data sharing with external entities through existing agreements between the medical centers and the homeless management information systems. A de-identified dataset can be requested of the corresponding author but will require executing additional data sharing agreements.

## Abbreviations

HMIS: Homeless Management Information System
PCORnet: Patient Centered Outcomes Research Network
ICD: International Classification of Diseases
ED: Emergency Department
IRB: Institutional Review Board
PRR: Prevalence Rate Ratio
HCUP: Healthcare Cost & Utilization Project
