# Supplementary material for: Variability in comorbidites and health services use across homeless typologies: multicenter data linkage between healthcare and homeless systems
Source: BMC Public Health. 2021 May 13;21:917. doi: 10.1186/s12889-021-10958-8 (PMC8117275; doi:10.1186/s12889-021-10958-8)
Supplement: Supplementary file 1 — Additional file 1. [file 12889_2021_10958_MOESM1_ESM.docx]

Additional file 1. Values used to generate Figure 2, including the point estimate and 95% CI.

|  | **No homeless services** | | **Homeless services,**  **no stable housing** | |
| --- | --- | --- | --- | --- |
| *Condition* | PRR | 95% CI | PRR | 95% CI |
| Hyperlipidemia | 1.1 | 0.9 to 1.3 | 0.7 | 0.6 to 0.8 |
| Hypertension | 1.3 | 1.2 to 1.4 | 0.9 | 0.8 to 1.0 |
| Diabetes | 1.3 | 1.1 to 1.5 | 0.9 | 0.7 to 1.0 |
| Asthma | 1.7 | 1.5 to 2.0 | 1.3 | 1.1 to 1.5 |
| Bipolar disorder | 2.0 | 1.7 to 2.4 | 1.5 | 1.3 to 1.8 |
| Depression | 2.2 | 1.9 to 2.5 | 1.4 | 1.3 to 1.6 |
| Anxiety | 2.5 | 2.1 to 3.0 | 1.4 | 1.1 to 1.6 |
| Cocaine abuse | 2.9 | 2.3 to 3.6 | 1.5 | 1.2 to 1.8 |
| Schizophrenia | 3.3 | 2.7 to 4.0 | 2.2 | 1.8 to 2.7 |
| Alcohol abuse | 4.4 | 3.6 to 5.3 | 2.0 | 1.6 to 2.4 |

Abbreviation: PRR=Prevalence Rate Ratio
