# Supplementary material for: Variability in comorbidites and health services use across homeless typologies: multicenter data linkage between healthcare and homeless systems
Source: BMC Public Health. 2021 May 13;21:917. doi: 10.1186/s12889-021-10958-8 (PMC8117275; doi:10.1186/s12889-021-10958-8)
Supplement: Supplementary file 2 — Additional file 2. [file 12889_2021_10958_MOESM2_ESM.docx]

Additional File 2. Conditions and associated ICD10 codes used to identify primary diagnoses recorded for emergency department visits.

| **Primary diagnoses, emergency visits** | **ICD10 Codes** |
| --- | --- |
| Prescription refill | Z76.0 |
| Chest pain, unspecified | R07.9 |
| Alcohol abuse with intoxication | F10.120; F10.129 |
| Administrative exam | Z02.9 |
| Suicidal ideation | R45.851 |
| Asthma, unspecified | J45.909 |
| Schizophrenia or auditory hallucinations | F20.0; F20.9 |
| Low back pain | M54.5 |
| Abdominal pain, unspecified | R10.9 |
| Foot pain | M79.671; M79.671 |
